# Polygenic score for C-reactive protein is associated with accelerated cortical thinning and increased psychopathology in adolescents: a population-based longitudinal cohort study

**DOI:** 10.21203/rs.3.rs-6823364/v1

**Published:** 2025-06-30

**Authors:** Haixia Zheng, Jonathan Savitz, Ebrahim Haroon, Jonathan Ahern, Robert Loughnan, Firas Nabber, Bohan Xu, Katherine Forthman, Robin Aupperle, Leanne Williams, Martin Paulus, Chun Chieh Fan, Wesley Thompson

**Affiliations:** Laureate Institute for Brain Research; Laureate Institute for Brain Research; Emory School of Medicine; University of California San Diego; Department of Cognitive Science, University of California, San Diego; Laureate Institute for Brain Research; Laureate Institute for Brain Research; Laureate Institute for Brain Research; Laureate Institute for Brain Research; Stanford University; Laureate Institute for Brain Research, Tulsa, USA; Laureate Institute for Brain Research; Laureate Institute for Brain Research

## Abstract

Adolescence is a critical neurodevelopmental window characterized by rapid cortical thinning, during which vulnerability to psychiatric disorders significantly increases. Although increased rates of cortical thinning have been associated with adverse mental health outcomes, the biological mechanisms underlying atypical neurodevelopment remain unclear. This study investigates whether genetic predisposition to systemic inflammation, assessed via polygenic scores for C-reactive protein (PGS_CRP), influences cortical thinning trajectories and psychopathology risk in adolescents. Using longitudinal data from the Adolescent Brain Cognitive Development (ABCD) Study (baseline n=11,214; follow-up n=7,823), we found that higher genetic susceptibility to inflammation was associated with accelerated cortical thinning, particularly in medial temporal and insular regions (β = −0.013 ~ −0.018, p.FDR < 0.05), and increased externalizing psychopathology symptoms (β = 0.167, p.FDR < 0.05). Early-life infections independently predicted greater depressive and externalizing symptoms (β = 0.511 ~0.608, p.FDR < 0.05) but did not interact significantly with genetic predisposition. Structural equation modeling revealed that cortical thinning partially mediated the relationship between genetic inflammation risk and externalizing symptoms. Moreover, neurobiological annotation showed regional overlaps between inflammation-linked cortical thinning and neurotransmitter receptor gradients involving serotonin, GABA, cannabinoid, and glutamate systems. These findings provide evidence for genetic predisposition to inflammation as a factor in adolescent cortical maturation and behavioral outcomes, highlighting potential neuroimmune mechanisms underpinning vulnerability to mental health disorders during this sensitive developmental period.

## Introduction

Adolescence is an important neurodevelopmental period during which rapid cortical thinning occurs^1,2^ and when many psychiatric disorders begin to emerge^3^. Aberrant patterns of cortical development, including accelerated thinning in frontal regions, are associated with worse cognitive and psychopathological outcomes in adolescents^4–8^, suggesting that deviation from typical neuroanatomical development captured by magnetic resonance imaging (MRI) indices of cortical thinning may partially underpin worse mental health outcomes in this sensitive age range. However, the upstream processes that drive atypical brain development remain poorly understood. Identifying the genetic and environmental factors–and their interactions–that shape neurodevelopmental trajectories is critical for understanding the origins of mental health disorders that often arise during the vulnerable stage of adolescence.

Emerging evidence suggests that immune signaling may play a key role in neurodevelopment. Cortical thinning, as measured by MRI, reflects key neurodevelopmental processes such as synaptic pruning, myelination, neurogenesis, and synaptogenesis^9–13^. These processes are highly susceptible to immune activation, which can result in lasting alterations in neural circuitry and brain function^12,14,15^. At a cellular level, immune activation influences cortical development via glial cells, such as astrocytes and microglia. These glial cells not only support neuronal health but also actively participate in synaptic pruning and neural circuit formation^16,17^. Inflammatory signals originating in the periphery can reach the brain via multiple pathways, including cytokine transport across the blood-brain barrier, vagal nerve signaling, and monocyte trafficking^18^, altering microglia activation^19^. These interactions also potentially disrupt neurotransmitter systems (e.g., such as serotonin, dopamine and glutamate), and perturb neurocircuits involved in mood regulation^18^. Indeed, neuroimaging studies have consistently show that inflammation is associated with disrupted brain circuits integral to motivation, emotion regulation, and cognitive processing^20^. Additionally, genetic and epigenetic studies have suggested that inflammation-related genetic profiles may influence risk for externalizing and internalizing psychopathology during childhood and adolescence by shaping neurocognitive development^5,21,22^.

C-reactive protein (CRP), an acute-phase protein synthesized by the liver, is one of the most widely studied peripheral markers of systemic inflammation. CRP levels correlate strongly with other inflammatory cytokines, including interleukin (IL)-6, IL-1β, and tumor necrosis factor^23–25^, and show high correspondence with cerebrospinal fluid CRP (r = 0.855)^26^, potentially explaining why peripheral CRP concentration has been associated with structural brain alterations^27,28^ and various psychiatric disorders, including major depressive disorder^29,30^, bipolar disorder^31^, and schizophrenia^32^. While circulating CRP levels reflect active inflammation at a specific time point, they are also influenced by non-specific factors, such as socio-economic disparities, age, body mass index, smoking, and sleep disturbance, making direct comparisons of inflammation challenging^33^. In contrast, the polygenic score for CRP (PGS_CRP)- a weighted sum based on an individual’s genotype that quantifies genetic predisposition for elevated CRP levels - provides a static estimate capturing an individual’s risk for inflammation. By capturing the heritable component of CRP levels, PRS_CRP (explaining approximately 16% of the variance in plasma CRP levels) is not confounded by transient environmental or behavioral influences^34^. Therefore, PGS_CRP provides an instrument which enables the investigation of how inherited proinflammatory tendencies influence brain development and psychiatric risk, and allows for the modeling of gene-by-environment interactions.

While gene by environment research has traditionally focused on psychosocial adversity, there is growing interest in biological stressors, particularly the burden of infections in childhood, may potentially contribute to later mental health outcomes. A large longitudinal study has shown that a high burden of childhood infections is associated with increased depressive symptoms in adolescence^35^. Building on this evidence, the present study primarily investigates the impact of genetic predisposition to elevated C-reactive protein (CRP) levels on adolescent neurodevelopment and psychopathology. We additionally explore whether early-life infection moderates this association, providing insight into potential gene-by-environment interactions. We hypothesized that: 1) greater genetic predisposition for elevated CRP levels is associated with an increased psychopathology risk and with altered patterns of cortical thinning; 2) genetic risk interact with early-life infection to influence the psychopathology outcomes and cortical thinning process; 3) neurobiological processes associated with cortical thinning partially mediate the relationship between genetic predisposition for high CRP levels and psychopathology. We also explore the potential neurobiological mechanisms underlying these hypothesized effects by examining whether the spatial pattern of cortical thinning shows a statistical correspondence with known distributions of neurotransmitter receptors.

## Methods

### Study Design and Population

This longitudinal cohort study utilized data from the Adolescent Brain Cognitive Development^SM^ (ABCD) Study, an ongoing large-scale study of n = 11,214 participants who were aged 9–10 years at baseline (see table 1 for the summary of demographic information). Participants were recruited from 21 sites across the United States. Data from both baseline (T_0_, n=11,214) and the two-year follow-up (T_2_, n=7,823) were included in the analyses. Demographic, clinical, and structural MRI data were obtained from the National Institutes of Mental Health Data Archive (NDA). Full recruitment details are available in previous publications^36,37^.

Exclusion criteria for the ABCD Study© included non-fluency in English, not having a guardian fluent in English or Spanish, major medical or neurological conditions, gestational age below 28 weeks or birthweight under 1,200 grams, contraindications for MRI scanning, a history of traumatic brain injury, current diagnosis of schizophrenia, moderate to severe autism spectrum disorder, intellectual disability, or alcohol/substance use disorder. Institutional review board approval was obtained for each participating site before data collection commenced.

This report was based on data from the ABCD 5.1 data release. The data analyzed were collected between September 1, 2016, and February 15, 2021. Written informed consent was provided by all parents, and assent was obtained from all children. This report follows the Strengthening the Reporting of Observational Studies in Epidemiology (STROBE) reporting guidelines.

### Exposure Variables

The primary exposure variable for this report was the PGS_CRP, which provides a quantitative instrument of an individual's genetic propensity for elevated systemic inflammation. The PGS_CRP was calculated as the weighted sum of genetic variants reached genome-wide significance (p < 5 × 10^−^⁸) for association with plasma CRP levels, using summary statistics from the UK Biobank participants (N = 427,367, European descent) and the Cohorts for Heart and Aging Research in Genomic Epidemiology (CHARGE) Consortium (total N = 575,531 European descent)^34^. Genetic data for the ABCD Study participants were collected, imputed using the TOPMED server, and processed using the Bayesian scoring method PRScs and PLINK 2.0 to generate the individual polygenic score for CRP. Further details on quality control and computational procedures are provided in the Supplementary Materials.

The secondary exposure variable was early-life infection, operationalized as a binary indicator based on parental reports of infant illness. Specifically, parents were asked: "How many days in the first 12 months of life did your child have any severe infections?" Due to the lack of detailed diagnostic information (e.g., infection type, severity, or duration), we applied a binary coding approach: responses indicating no reported sick days were coded as 0 (no severe early-life infection), and those reporting one or more days of illness were coded as 1 (presence of severe early-life infection). Although limited in clinical detail, this measure provides a proxy for early-life infection as a biological stressor.

### Outcome Variables

The primary outcomes of interest in this study were MRI indices of cortical thickness and measures of psychopathology. The MRI acquisition and processing procedures adhered to protocols established by the ABCD Study, described in detail elsewhere^36,37^. Briefly, imaging data were obtained from three types of 3T MRI scanners (Siemens Prisma/Prisma Fit, General Electric MR 750, Philips Achieva dStream/Ingenia). T1-weighted images underwent correction for gradient nonlinearity distortions using scanner-specific nonlinear transformations. Cortical reconstruction and volumetric segmentation were performed using FreeSurfer v7.1.1 with processing pipelines designed to address common issues in MRI data, such as head motion, distortion, and intensity inhomogeneity^36^. Cortical thickness was mapped to 34 parcellations per hemisphere based on the Desikan–Killiany atlas^38^. The ABCD Data Analysis, Informatics, and Resource Center (DAIRC) conducted both automated and manual quality control reviews before sharing the data.

Psychopathology was assessed using the depression, internalizing and externalizing symptom scales from the parent-reported Child Behavior Checklist (CBCL). The CBCL is a well-validated and widely used tool for evaluating youth mental health, known for its high reliability and internal consistency^39^. It consists of eight syndrome scales (anxious, depressed, somatic complaints, social problems, thought problems, attention problems, rule-breaking behavior, and aggressive behavior) that load into two broad categories: internalizing and externalizing problems^40^. The internalizing and externalizing scores from CBCL are commonly used for identifying broad patterns of emotional and behavioral problems^40^. Depression was examined as a distinct outcome due to its connection inflammatory markers in the literature and its central role in mental health disorders^41^. The continuous estimation of depression was derived from CBCL DSM-5 oriented affective problem scale, developed in the 2001 revision of the CBCL. Parents rated the presence of specific child behaviors over the past six months using a scale of 0 (“Not True”), 1 (“Somewhat or Sometimes True”), or 2 (“Very True or Often True”), with higher scores representing more significant psychopathology.

### Covariates

Sex at birth, body mass index (BMI), self-reported race/ethnicity, parental education, household income, and the first ten principal components of genetic ancestry (to account for population stratification) were included as fixed-effect covariates in analyses. These covariates have been previously reported to be associated with many environmental and genetic-related exposures and health outcomes. Parental education and household income were reported by the guardian and are key indicators of socioeconomic status, known to be associated with neurodevelopmental trajectories and psychopathology^42,43^. The ten principal components of genetic ancestry were derived from the ABCD genetic data to account for population stratification across diverse ancestral backgrounds, thereby minimizing spurious associations unrelated to the exposure and outcomes of interest. The detailed methodology for computing these genetic principal components has been previously published^44^. Together, these covariates were included to control for confounds and to help isolate the unbiased effects of the primary exposure variables on the outcomes of interest.

Additionally, individual, family, and study site were included as random-effect covariates in the analyses to account for nesting of the data with respect to these variables. Individual random effects account for nesting of participants’ repeated measurement occasions (two visits). Many ABCD Study participants come from families with siblings also in the study (9420 families); hence, family random effects account for nesting of data within families. Finally, there are 21 data collection sites in the ABCD Study, and site random intercepts account for nesting at this level.

### Statistical Analyses

Participants were stratified by genetic ancestry into two groups to account for population stratification: Sample 1, individuals of European ancestry (EU Sample, N = 6,605 at baseline and N = 5,992 at follow-up), and Sample 2, individuals of non-European ancestry (Non-EU Sample, N = 4,500 at baseline and N = 3,750 at follow-up). This stratification was conducted to mitigate bias arising from population stratification, a phenomenon where differences in allele frequencies across ancestral groups reflect demographic history rather than trait-specific associations^45^. Given that genetic variants contributing to polygenic scores can vary in frequency and effect size between populations, stratification ensures that the polygenic scores reliably capture ancestry-specific genetic risk, thereby enhancing the validity and generalizability of the findings^45^.

A meta-analysis was conducted to integrate findings across participants of European and non-European ancestry samples. This approach was chosen to synthesize results from diverse genetic backgrounds, enhancing the generalizability of our findings and providing a robust assessment of the genetic influences on neurodevelopmental outcomes across different populations. Statistical significance was evaluated using a False Discovery Rate (FDR) threshold of P < 0.05 of meta-analysis results.

Linear mixed-effects models (LMEs) were used to examine whether genetic predisposition to systemic inflammation, as indexed by the PGS_CRP, was associated with cortical thickness trajectories and psychopathology risk during adolescence (primary hypothesis), and whether this association was moderated by early-life infection (secondary hypothesis). The model included a three-way interaction between PGS_CRP, age, and early-life infection, with age modeled continuously to capture longitudinal change from baseline (T0) to follow-up (T2).

The model was specified as:

$$\text{Outcome}s_{\text{ijk}}=\beta_{0}+\beta_{1}(\text{Ag}e_{\text{ijk}})+\beta 2(\text{PGS}\_\text{CR}P_{i})+\beta 3(\text{Ag}e_{\text{ijk}}\times \text{PGS}\_\text{CR}P_{i})+\beta 4(\text{EarlyLifeInfectio}n_{i})+\beta 5(\text{Ag}e_{\text{ijk}}\times \text{EarlyLifeInfectio}n_{i})+\beta 6(\text{PGS}\_\text{CR}P_{i}\times \text{EarlyLifeInfectio}n_{i})+\beta 7(\text{Ag}e_{\text{ijk}}\times \text{PGS}\_\text{CR}P_{i}\times \text{EarlyLifeInfectio}n_{i})+X_{\text{ijk}}\beta +u_{i}+v_{j}+w_{k}+\varepsilon_{\text{ijk}}$$

where i, j, and k index individuals, families, and study sites, respectively. X includes fixed-effect covariates: sex, body mass index (BMI), race, parental education, household income, and the first ten principal components of genetic ancestry. The random effects including individual ID (*u*_*i*_), family ID (*v*_*j*_), and study site (*w*_*k*_). The *ε*_*ijk*_ represents the residual error. All continuous predictors were standardized prior to analysis.

### Mediation Analyses

We implemented structural equation models (SEMs) to examine whether changes in cortical thickness mediate the association between PGS_CRP and psychopathology. SEMs included depressive, internalizing, and externalizing symptoms at 2-year follow-up as outcome variables; each was regressed on changes in global mean cortical thickness (T_2_ – T_0_), PGS_CRP, age at T2, sex, baseline cortical thickness, and baseline psychopathology symptoms. Global mean cortical thickness change was included as a mediator of the PGS_CRP to psychopathology relationship. Although primary analyses tested regional thickness associations, global cortical thinning was used in the mediation models to capture a unified index of brain maturation. This is supported by cortical thinning during adolescence is a global coordinated process reflecting normative neurodevelopment. Using global thickness change provides a developmentally meaningful and statistically efficient summary of individual differences in cortical maturation. One-thousand bootstrap samples were used to obtain robust estimates and confidence intervals for direct and indirect effects. Model fit was evaluated using the comparative fit index (CFI), Tucker-Lewis index (TLI), and standardized root mean square residual (SRMR).

### Biological Annotation

To explore the biological mechanisms underlying the effects of PGS_CRP on cortical thinning in adolescents, we utilized the Neuromaps toolbox^46^ for biological annotation. Our neuroimaging results were aligned and compared to standardized neurotransmitter receptor density maps, derived from PET tracer studies covering 17 receptors and transporters across nine neurotransmitter systems. These include serotonin (5-HT_1A_^47^, 5-HT_1B_^47^, 5-HT_2A_^48^, 5-HT_4_^48^, 5-HT_6_^49^, 5-HTT^48^), cannabinoid (CB_1_^50^), dopamine (D_1_^51^, D_2_^52^, DAT^53^), gamma-aminobutyric acid type a receptor (GABA_A_^54^), histamine (H_3_^55^), norepinephrine (NET^56^), acetylcholine (M_1_^57^, VAChT^58^), glutamate (mGluR_5_^59^), and opioid (MOR^60^). Spatial-autocorrelation-preserving permutation tests, termed spatial null model^46,61^, were employed to statistically assess the similarity between our neuroimaging findings and these molecular signatures, providing insights into the specific molecular and cellular mechanisms that may mediate the impact of PGS_CRP on the development of psychopathological symptoms.

## Results

### PGS_CRP Effects on Cortical Thinning and Psychopathology

**Figure 1:**
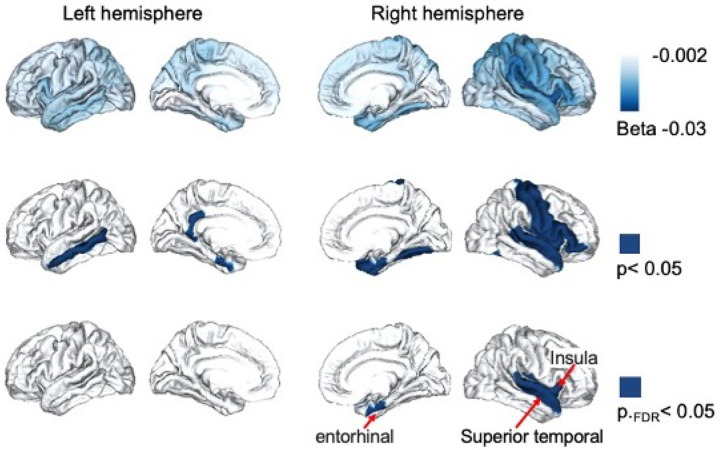
PGS_CRP is associated with variations in age-related cortical thinning in Adolescence, with strongest acceleration effect in the medial temporal lobe. Cortical thickness naturally decreases with maturation. This figure illustrates the interaction between polygenic scores for C-reactive protein (PGS_CRP) and age, showing how genetic predisposition to inflammation influences the trajectory of cortical thinning during adolescence. The top row shows the effect sizes (β3) for the interaction term (Age × PGS_CRP), with darker blue indicating stronger associations with accelerated cortical thinning throughout the brain. The middle row highlights regions where the interaction term was significant at meta-analysis p<0.05), and the bottom row identifies regions that meta-analysis results passed the false discovery rate (FDR) threshold (*p*FDR<0.05).

The interaction term (*β*_*3*_) allowed us to determine whether genetic predisposition for systemic inflammation, as captured by PGS_CRP, modifies age-related cortical thinning. Meta-analytic findings across ancestry groups revealed that higher PGS_CRP was significantly associated with accelerated cortical thinning throughout brain cortex and particularly in medial temporal regions (Figure 1). As shown in Figure 1, three regions passed our statistical threshold (meta-analysis *p*.FDR <0.05), including the right entorhinal cortex (*β*_*3*_ = −0.016, SE = 0.005, *p*.FDR < 0.05), right insula cortex (*β*_*3*_ = −0.018, SE = 0.005, *p*.FDR < 0.05), and right superior temporal gyrus (*β*_*3*_ = −0.013, SE = 0.004, *p*.FDR < 0.05). These findings suggest that individuals in ABCD with a higher genetic risk for systemic inflammation experience steeper cortical thinning trajectories in these regions over time. Regions that survived FDR correction at are presented in Table 2, while the full set of results is available in **Supplementary Table 1**.

We observed a significant association between the PGS_CRP scores and externalizing psychopathology. Specifically, higher PGS_CRP scores were associated with greater externalizing symptoms, including behavioral problems such as aggression or rule-breaking (*β*_*2*_ = 0.167, SE = 0.069, *p*.FDR = 0.048). This suggests that genetic predisposition for elevated inflammation may contribute to behavioral dysregulation during adolescence. No significant PGS_CRP effects on depression or internalizing psychopathology. The full set of results is available in **Supplementary Table 2**.

### Early-Life Infection Effects on Cortical Thinning and Psychopathology

Meta-analysis showed no significant effects of early-life infection on cortical thickness, nor were there significant interaction effects of early-life infection and Age or PRS_CRP on cortical thickness. However, individuals with a reported early-life infection exhibited significantly higher depression scores (*β*_*2*_ = 0.511, SE = 0.232, p.FDR = 0.042) and elevated externalizing psychopathology (*β*_*2*_ = 0.589, SE = 0.232, p.FDR = 0.034). The association with internalizing psychopathology was not significant (*β*_*2*_ = 0.608, SE =0.363, p.FDR = 0.094). Detailed results are available in **Supplementary Table 3.**

### Mediation pathways

The SEM demonstrated excellent fit: CFI = 1.00, TLI = 1.00, and SRMR = 0.000, indicating no significant misfit.

**Figure 2:**
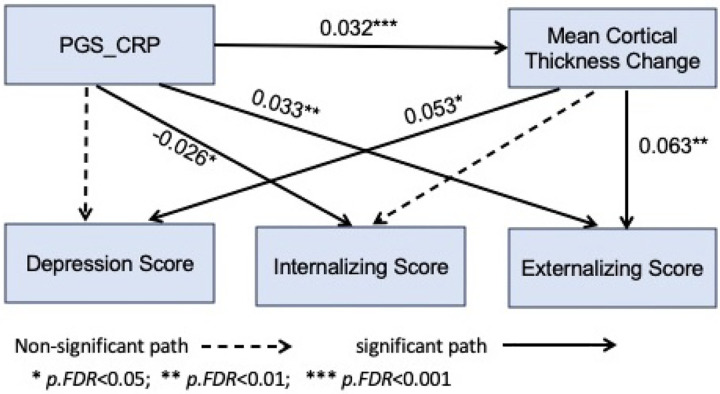
Path diagram illustrates the association between PGS_CRP and externalizing psychopathology, which was partially mediated by mean cortical thickness change. The pathway demonstrates that individuals with a higher polygenic score for CRP tend to experience accelerated cortical thinning, which is associated with increased externalizing psychopathology. However, the mediation is partial (4% of total effect), as PRS_CRP also has a direct effect on externalizing psychopathology, indicating that the behavioral outcomes are not solely explained by cortical thinning.

As demonstrated in Figure 2, changes in global mean cortical thickness were associated with depressive symptoms (*b* = 0.053, *p*.FDR = 0.010) and externalizing symptoms at T_2_ (*b* = 0.063, *p*.FDR = 0.002), but not with internalizing symptoms (*b* = 0.040, *p*.FDR = 0.051). PGS_CRP was negatively associated with internalizing symptoms at T_2_ (*b* = −0.026, *p*.FDR = 0.043) but positively associated with externalizing symptoms (*b* = 0.033, *p*.FDR = 0.008). No significant associations were found between PGS_CRP and depressive symptoms (*b* = −0.0005, *p*.FDR = 0.966).

The indirect (mediation) effect of PGS_CRP on externalizing symptoms (*b* = 0.002, *p*.FDR = 0.014) and depression (*b* = 0.002, *p*.FDR = 0.035) through cortical thickness was significant, while the indirect effect on internalizing symptoms was not (*b* = 0.001, *p*.FDR = 0.072). Total effect estimates were significant between PGS_CRP and externalizing symptoms (*b* = 0.035, *p*.FDR = 0.005) but not internalizing symptoms (b = −0.024, *p*.FDR = 0.051) and depressive symptoms (b = 0.001, *p*.FDR = 0.966). Approximately 4% of the total effect of PGS_CRP on externalizing psychopathology was mediated through the change in cortical thickness. The detailed results are available in **Supplementary Table 4.**

### Biological annotation

**Figure 3:**
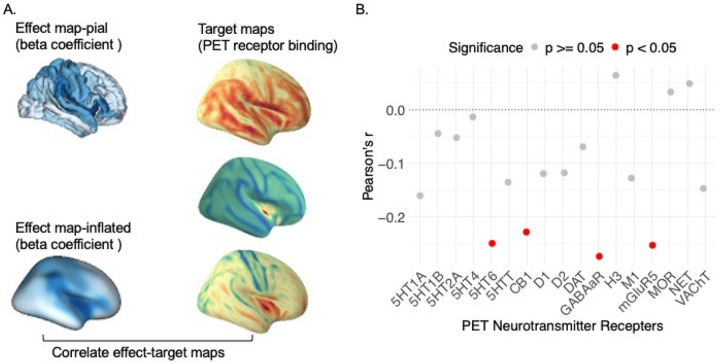
Regional Association Between PGS_CRP Effects on Cortical Thinning and Neurotransmitter Receptor Gradients. (A) The left panel displays the regional effect map of PGS_CRP on cortical thinning (PGS_CRP by age interaction effect beta coefficients) mapped onto the cortical surfaces. The target maps on the right show neurotransmitter receptor distributions based on positron emission tomography (PET) receptor binding for corresponding regions. These neurotransmitter maps are included to aid in conceptualizing receptor gradients across regions and understanding the process; the colors are not indicative of specific data values or quantitative comparisons. (B) The scatter plot in the right panel depicts Pearson’s correlation coefficients (r) between the PGS_CRP effect on cortical thinning and the gradients of various PET neurotransmitter receptors. Red dots indicate significant correlations (*p*_*uncorrected*_ < 0.05), with notable associations observed for serotonin (5HT_6_), gamma-aminobutyric acid type a receptor (GABAaR), cannabinoid (CB_1_), and metabotropic glutamate receptor 5 (mGluR5).

We tested whether the regional effects of PGS_CRP on cortical thinning exhibited any correspondence with neurotransmitter receptor gradients. To explore this, we performed correlations between the effect map of PGS_CRP on cortical thinning and maps of various neurotransmitter receptor distributions. As showed in Figure 3, our analysis revealed significant correlations between PGS_CRP effects and four specific neurotransmitter receptor gradients: serotonin (5HT_6_, r = −0.250, *p*_uncorrected_ = 0.010, p.FDR=0.085), gamma-aminobutyric acid type a receptor (GABAaR, r = −0.274, *p*_uncorrected_ = 0.010, p.FDR=0.085), cannabinoid (CB_1_, r = −0.228, *p*_uncorrected_ = 0.020, p.FDR=0.085), and metabotropic glutamate receptor 5 (mGluR5, r = −0.253, *p*_uncorrected_ = 0.020, *p*.FDR=0.085). Although these associations did not survive FDR correction, it highlighted that the regional effects of PGS_CRP on cortical thinning are possibly non-randomly distributed and align with neurotransmitter receptor gradients, emphasizing the role of immune-neurobiological pathways in cortical maturation and their potential contribution to psychopathology susceptibility. The full set of results is available in **Supplementary Table 5**.

### Sensitivity Analysis

To ensure the robustness of our findings, we conducted a sensitivity analysis by combining the European (EU) and non-European (non-EU) samples into a single full-sample analysis, rather than separating them and performing a meta-analysis. The results of the full-sample analysis were consistent with the meta-analytic approach, demonstrating similar directional effects across all primary outcomes. Notably, beyond the previously identified PGS_CRP by Age interaction effect in the right entorhinal cortex, right insula cortex, and right superior temporal gyrus, the combined sample revealed eight additional subregions that passed the statistical threshold of *p*.FDR < 0.05, reflecting the increased statistical power and precision afforded by the larger sample size (Detailed results are provided in **Supplementary Table 6**). Furthermore, the association between PGS_CRP and externalizing psychopathology remain statistically significant (Detailed results are provided in **Supplementary Table 7**). All associations between early-life infection and psychopathology remained statistically significant (Detailed results are provided in **Supplementary Table 8**). These sensitivity analyses all yield stronger effects than the primary analyses, highlights that the observed associations are robust and not dependent on the specific analytical strategy employed.

## Discussion

The primary goal of this study was to examine how genetic predisposition for systemic inflammation, as measured by polygenic scores for C-reactive protein (PGS_CRP), influences adolescent neurodevelopment and the emergence of psychopathology, and explore its neurobiological mechanisms. Consistent with our initial hypotheses, higher genetic susceptibility to inflammation was associated with accelerated cortical thinning, particularly in medial temporal and insular regions, as well as increased externalizing psychopathology symptoms. Notably, we identified a significant indirect pathway wherein cortical thinning partially mediated the relationship between genetic predisposition to inflammation and both externalizing behaviors and depressive symptoms. Biological annotation analyses further suggested that the regions affected by PGS_CRP-related cortical thinning overlapped with neurotransmitter receptor systems enriched in serotonin, GABA, cannabinoid, and glutamate signaling, highlighting the potential role of inflammation in disrupting these neurobiological pathways. These results underscore the importance of systemic inflammation as a key factor influencing neurodevelopmental trajectories during adolescence. By linking genetic predisposition for inflammation to structural brain changes and behavioral outcomes, this study provides a foundation for future research aimed at understanding and mitigating the risks associated with inflammation-driven alterations in brain development and psychopathology.

Accelerated cortical thinning during adolescence has been linked to a range of psychiatric outcomes. In schizophrenia, for example, progressive cortical thinning, particularly in the frontotemporal cortex, is associated with symptom severity and illness duration, from the first episode of psychosis through to the chronic stages of the disorder^62,63^. Similarly, individuals at high genetic risk for bipolar disorder show accelerated thinning and volume reduction in frontal regions, even before the onset of the disorder^64^. Extending these findings, our study suggesting that genetic predisposition for inflammation, as measured by PGS_CRP, maybe a critical influencer of cortical developmental trajectories. This aligns with previous evidence linking cortical thinning during maturation to gene expression related to dendrites, dendritic spines, and myelin, suggesting that shared molecular pathways may underlie both cortical development and psychiatric vulnerability^12,65,66^. In our study, the brain regions most affected by accelerated thinning—such as the entorhinal cortex, superior temporal gyrus, and insula—play key roles in integrating emotional and cognitive information. The superior temporal gyrus is crucial for auditory and language comprehension^67^, the insula detects relevant stimuli and coordinates neural resources for emotional and cognitive processing^68^, and the entorhinal cortex is essential for encoding and retrieving emotional memories^69^. Moreover, the relationship between inflammation and cortical thinning appears to converge on molecular pathways involving complement component proteins like C4A. These proteins, known for their role in synaptic pruning, have been linked to similar brain structural alterations (i.e., insula and entorhinal cortex) and neurocognitive outcomes in both adolescent and adults samples^70,71^. These findings are also consistent with neuroimaging research showing that inflammation disrupts neural circuits involved in emotional regulation and cognition^20^, thus supporting a potential neurobiological mechanism by which inflammation-related genetic risk factors influence psychiatric outcomes^5,21,22^.

Building on these findings, the association between PGS_CRP and externalizing psychopathology is significant. Externalizing behaviors, including aggression and rule-breaking, are often linked to deficits in emotion regulation and impulse control. Our results suggest that systemic inflammation, driven by genetic factors, may impair the maturation of brain regions necessary for regulating behavior, increasing the risk for externalizing psychopathology. Importantly, these findings do not contradict the existing literature. While previous studies have primarily linked elevated inflammatory markers with fatigue, depression, and stress-related disorders^18,27,41,72^—commonly associated with energy-conserving 'sickness behavior'— our results expand this understanding by implicating systemic inflammation in poor decision-making and impulsive behaviors, which heighten the risk for externalizing psychopathology. Furthermore, we observed a significant increase in the association between PGS_CRP and depression scores in the non-European ancestry sample (**Supplementary Table 2**), consistent with prior research. This discrepancy is likely attributable to our stringent statistical approach, which prioritizes robust and conservative estimates. Additionally, it is important to note that the ABCD cohort primarily consists of healthy adolescents without clinical depression, which may further attenuate associations between PGS_CRP and depression scores.

Contrary to our hypotheses, early-life infection did not interact significantly with genetic risk to affect cortical thinning trajectories. However, early-life infection independently predicted increased risk for depressive and externalizing symptoms, reinforcing prior evidence that early immunological challenges can have lasting impacts on mood disorders^73^. Our data supports the "dual-hit" hypothesis^74^, where early environmental insults, such as infections, interact with genetic predispositions to heighten the risk for psychopathology later in life. The impact of early immune activation on neurodevelopment may sensitize the brain immune cell microglia (microglia priming)^75^ to subsequent inflammatory stimuli, further disrupting normal synaptic pruning or other maturation processes. The absence of a significant interaction with genetic predisposition could reflect limitations in statistical power or distinct biological pathways through which early-life infections and genetic risk independently contribute to psychopathology. Additionally, our reliance on parental reporting of early-life infections introduces the possibility of recall bias and lacks detailed information regarding infection source, severity, and duration. Future studies should incorporate more objective and detailed measures of early-life infection—including clinical diagnoses, infection severity, duration, and specific pathogens—to improve our understanding of how these factors interact with genetic inflammation susceptibility to influence neurodevelopmental trajectories.

The biological annotations provide deeper insights into the neurobiological mechanisms at play. Specifically, we found a significant overlap between regions of cortical thinning associated with PGS_CRP and neurotransmitter receptor gradients for serotonin, GABA, cannabinoid, and glutamate signaling. This aligns with decades of animal research showing that inflammation critically modifies neurotransmitter systems, thereby increasing the risk of psychiatric disorders^74,76^. Notably, while inflammation and disruptions in serotonin and glutamate signaling have been extensively studied in relation to "sickness behaviors" like fatigue and depression^77–80^, our findings extend this perspective. Our findings, while preliminary, indicate potential neurochemical pathways through which genetic predisposition for inflammation may alter cortical development, contributing to externalizing psychopathology, which includes deficits in emotion regulation, poor impulse control, and impaired decision-making.

Our findings have important clinical implications, highlighting genetic predispositions to systemic inflammation as key factors in adolescent brain development and psychopathology, opening avenues for early intervention. Targeting inflammation in at-risk individuals could help prevent psychopathology through anti-inflammatory treatments or lifestyle interventions like exercise and dietary changes. Early identification of children with higher genetic liability for systemic inflammation may also help clinicians identify those at higher risk for neurodevelopmental disorders or psychiatric symptoms. However, several limitations of this study must be acknowledged. While we employed a polygenic score for CRP to assess genetic predisposition for inflammation, it explains only a fraction of the variance in plasma CRP levels, limiting its comprehensiveness. Additionally, the CRP GWAS used to derive the polygenic score was based on data from the UK Biobank, a predominantly adult cohort, which may not fully capture the genetic architecture of CRP during adolescence, potentially affecting the accuracy and predictability of the score for the ABCD cohort. Moreover, the lack of longitudinal measures of circulating CRP may have restricted our ability to capture the dynamic relationship between systemic inflammation and neurodevelopment. Future research should explore the interplay between genetic risk for inflammation and environmental pro-inflammatory factors—such as early-life adversity, viral infections, or diet—to better understand their contributions to neurodevelopmental trajectories.

## Conclusion

In conclusion, our findings revealed genetic predisposition to systemic inflammation, as quantified by polygenic scores for CRP, as a critical factor influencing adolescent neurodevelopmental trajectories and psychopathology risk. Specifically, elevated genetic susceptibility to inflammation was robustly associated with accelerated cortical thinning, particularly in regions vital for emotional and cognitive processing, and increased externalizing behavioral symptoms. Importantly, we identified cortical thinning as a partial mediator of the link between inflammation-related genetic risk and psychopathology, highlighting a tangible neurodevelopmental pathway through which genetic predispositions can manifest in behavioral dysfunction. While early-life infections independently heightened psychopathological risks, their interaction with genetic predisposition requires further nuanced investigation with detailed clinical data. Additionally, exploratory biological annotation suggested that disruptions of neurotransmitter receptor systems (serotonin, GABA, cannabinoid, and glutamate) may underpin these inflammation-linked cortical changes. Collectively, these findings highlight systemic inflammation as a potential target for early identification and preventive interventions in vulnerable youth, offering critical insights into the complex neuroimmune mechanisms that contribute to mental health disorders emerging during adolescence. Future studies should build upon these results by integrating detailed longitudinal measures of inflammation, environmental exposures, and precise mechanistic investigations to deepen our understanding of these critical developmental pathways.

## Supplementary Files

This is a list of supplementary files associated with this preprint. Click to download.

- supplement.docx

## Figures and Tables

**Table 1.** Demographic, exposure, and outcome variables used in current study.

All samples	Year0	Year2
n	11214	7823
Mean Cortical Thickness (mean [SD])	2.72 (0.08)	2.69 (0.08)
Depression (mean [SD])	53.60 (5.72)	53.78 (5.95)
Internalizing (mean [SD])	48.48 (10.66)	47.75 (10.53)
Externalizing (mean [SD])	45.77 (10.34)	44.63 (9.87)
PGS_CRP (mean [SD])	0.00 (1.00)	−0.01 (1.00)
Early-life Infection=Yes(%)	1710 (16.7)	1218 (16.9)
Age (mean [SD])	9.91 (0.63)	11.95 (0.65)
Sex = female (%)	5329 (47.5)	3591 (45.9)
BMI (mean [SD])	18.73 (3.96)	20.49 (4.45)
Race (%)		
White	5924 (52.8)	4306 (55.5)
Black	1670 (14.9)	1050 (13.5)
Hispanic	2217 (19.8)	1459 (18.8)
Asian	213 (1.9)	130 (1.7)
Multiracial	1188 (10.6)	816 (10.5)
Parental Education (%)		
Less than high school	513 (4.6)	334 (4.3)
High school diploma	1027 (9.2)	642 (8.3)
Some college	2910 (26.1)	1986 (25.7)
Bachelor	2866 (25.7)	2051 (26.6)
Post Graduate Degree	3829 (34.4)	2700 (35.0)
Household Income (%)		
[<50K]	3008 (29.3)	2013 (28.1)
[>=50K & <100K]	2930 (28.5)	2147 (29.9)
[>=100K]	4339 (42.2)	3009 (42.0)
**EU ancestry sample**	**Year0**	**Year2**
n	6336	4617
Mean Cortical Thickness (mean [SD])	2.74 (0.08)	2.70 (0.08)
Depression (mean [SD])	53.60 (5.66)	53.94 (6.02)
Internalizing (mean [SD])	48.70 (10.45)	48.30 (10.33)
Externalizing (mean [SD])	45.42 (10.01)	44.47 (9.65)
PGS_CRP (mean [SD])	−0.18 (0.93)	−0.18 (0.93)
Age (mean [SD])	9.92 (0.63)	11.96 (0.65)
Sex = female (%)	2982 (47.1)	2080 (45.1)
BMI (mean [SD])	17.92 (3.31)	19.69 (4.01)
Race (%)		
White	5807 (91.7)	4225 (91.9)
Black	3 (0.0)	2 (0.0)
Hispanic	270 (4.3)	182 (4.0)
Asian	4 (0.1)	2 (0.0)
Multiracial	251 (4.0)	185 (4.0)
Parental Education (%)		
Less than high school	37 (0.6)	31 (0.7)
High school diploma	221 (3.5)	149 (3.2)
Some college	1208 (19.1)	864 (18.8)
Bachelor	1967 (31.1)	1445 (31.5)
Post Graduate Degree	2892 (45.7)	2100 (45.8)
Household Income (%)		
[<50K]	780 (12.9)	566 (12.9)
[>=50K & <100K]	1856 (30.8)	1404 (32.0)
[>=100K]	3393 (56.3)	2424 (55.2)
**Non-EU ancestry sample**	**Year0**	**Year2**
n	4878	3206
Mean Cortical Thickness (mean [SD])	2.70 (0.08)	2.66 (0.08)
Depression (mean [SD])	53.61 (5.80)	53.55 (5.83)
Internalizing (mean [SD])	48.19 (10.91)	46.96 (10.76)
Externalizing (mean [SD])	46.26 (10.74)	44.81 (10.18)
PGS_CRP (mean [SD])	0.25 (1.04)	0.24 (1.03)
Age (mean [SD])	9.90 (0.62)	11.94 (0.66)
Sex = female (%)	2347 (48.1)	1511 (47.1)
BMI (mean [SD])	19.78 (4.46)	21.66 (4.80)
Race (%)		
White	117 (2.4)	81 (2.6)
Black	1667 (34.2)	1048 (33.1)
Hispanic	1947 (39.9)	1277 (40.3)
Asian	209 (4.3)	128 (4.0)
Multiracial	973 (19.2)	631 (19.9)
Parental Education (%)		
Less than high school	476 (9.9)	303 (9.7)
High school diploma	806 (16.7)	493 (15.8)
Some college	1702 (35.3)	1122 (35.9)
Bachelor	899 (18.7)	606 (19.4)
Post Graduate Degree	973 (19.4)	600 (19.2)
Household Income (%)		
[<50K]	2228 (52.4)	1447 (52.1)
[>=50K & <100K]	1074 (25.3)	743 (26.8)
[>=100K]	946 (22.3)	585 (21.1)

**Table 2:** Brain Regions with Significant PGS_CRP by Age Interaction Effects on Cortical Thinning

Regions	Data Source^1^	Beta^2^	SE^3^	*p*	*p* ^FDR^
right entorhinal cortex	Meta Analysis	−0.016	0.005	0.002	0.049
**right entorhinal cortex**	**EU Sample**	−**0.019**	**0.007**	**0.008**	**0.493**
right entorhinal cortex	Non-EU Sample	−0.014	0.008	0.098	0.308
**right insula cortex**	**Meta Analysis**	−**0.018**	**0.005**	**0.001**	**0.049**
right insula cortex	EU Sample	−0.016	0.007	0.028	0.493
right insula cortex	Non-EU Sample	−0.021	0.008	0.010	0.104
right superior temporal gyrus	Meta Analysis	−0.013	0.004	0.002	0.049
**right superior temporal gyrus**	**EU Sample**	−**0.012**	**0.005**	**0.024**	**0.493**
right superior temporal gyrus	Non-EU Sample	−0.015	0.007	0.026	0.139

1. Participants were stratified by genetic ancestry into two groups to address population stratification: individuals of European ancestry (EU Sample) and non-European ancestry (Non-EU Sample). Linear mixed-effects (LME) models were performed separately in each group, and a meta-analysis was conducted to integrate findings across both groups. Statistical significance was determined through meta-analysis, with a threshold of P < 0.05 after False Discovery Rate (FDR) correction.

2. Beta coefficient of PGS_CRP by age interaction effect on cortical thickness.

3. SE: Standard Error.
